# Genetic variant classification by predicted protein structure: A case study on IRF6

**DOI:** 10.1016/j.csbj.2024.01.019

**Published:** 2024-02-03

**Authors:** Hemma Murali, Peng Wang, Eric C. Liao, Kai Wang

**Affiliations:** a Graduate Program in Biochemistry and Molecular Biophysics, University of Pennsylvania, Philadelphia, PA 19104, United States; b Center for Cellular and Molecular Therapeutics, Children’s Hospital of Philadelphia, Philadelphia, PA 19104, United States; c Master of Biotechnology Program, University of Pennsylvania, Philadelphia, PA 19104, United States; d Department of Surgery, Perelman School of Medicine, University of Pennsylvania, Philadelphia, PA 19104, United States; e Center for Craniofacial Innovation, Children’s Hospital of Philadelphia, Philadelphia, PA 19104, United States; f Department of Pathology and Laboratory Medicine, University of Pennsylvania, Philadelphia, PA 19104, United States

**Keywords:** Variant interpretation, Structural biology, Clinical genetics

## Abstract

Next-generation genome sequencing has revolutionized genetic testing, identifying numerous rare disease-associated gene variants. However, to impute pathogenicity, computational approaches remain inadequate and functional testing of gene variant is required to provide the highest level of evidence. The emergence of AlphaFold2 has transformed the field of protein structure determination, and here we outline a strategy that leverages predicted protein structure to enhance genetic variant classification. We used the gene *IRF6* as a case study due to its clinical relevance, its critical role in cleft lip/palate malformation, and the availability of experimental data on the pathogenicity of *IRF6* gene variants through phenotype rescue experiments in *irf6*^*-/-*^ zebrafish. We compared results from over 30 pathogenicity prediction tools on 37 *IRF6* missense variants. *IRF6* lacks an experimentally derived structure, so we used predicted structures to explore associations between mutational clustering and pathogenicity. We found that among these variants, 19 of 37 were unanimously predicted as deleterious by computational tools. Comparing *in silico* predictions with experimental findings, 12 variants predicted as pathogenic were experimentally determined as benign. Even with the recently published AlphaMissense model, 15/18 (83%) of the predicted pathogenic variants were experimentally determined as benign. In comparison, mapping variants to the protein revealed deleterious mutation clusters around the protein binding domain, whereas N-terminal variants tend to be benign, suggesting the importance of structural information in determining pathogenicity of mutations in this gene. In conclusion, incorporating gene-specific structural features of known pathogenic/benign mutations may provide meaningful insights into pathogenicity predictions in a gene-specific manner and facilitate the interpretation of variant pathogenicity.

## 1 Introduction

Advancements in next-generation genome sequencing have revolutionized the use of genetic testing in clinical settings [1], [2], [3], [4], [5], spurred the growth of precision medicine [6], [7], [8], [9]], and led to the identification of numerous rare disease-associated genetic variants [10]. However, these developments have simultaneously brought about new challenges in interpreting and assigning clinical significance to these variants [11]. To address the complex challenge of variant interpretation, the American College of Medical Genetics and Association for Molecular Pathology (ACMG/AMP) established a set of guidelines[12,13] that recommends the incorporation of structural, population, functional, and clinical data for variant interpretation. These guidelines categorize variants into five levels of disease risk: Pathogenic (P), Likely Pathogenic (LP), Likely Benign (LB), Benign (B), and lastly, Uncertain Significance.

Variants of Uncertain Significance (VUS) are variants that are neither definitively pathogenic (disease-causing) nor definitively benign (harmless). The human genome is immensely diverse and contains countless genetic variations – naturally, not all have been thoroughly studied or linked to specific health conditions yet. VUS represent a temporary state of uncertainty in genetics and are subject to reclassification as our understanding grows with ongoing research and data accumulation. As of now, these large sets of unclassified VUS present a major obstacle in clinical genetics [14], [15], [16] since their presence often prolongs the diagnostic journey, causes diagnostic ambiguity and patient anxiety, and hinders our understanding of genetic impact on disease progression and presentation.

Further classifying VUS as pathogenic or benign in an accurate and efficient manner is crucial to expedite diagnosis, treatment, and enhance our understanding of their role in disease progression. Addressing the challenge of classifying VUS involves a multifaceted approach, beginning with collecting more genetic and clinical data, conducting functional studies, and improving computational tools. Functional studies of VUS are often time-consuming, resource-intensive, and not suitable for front-line assessment of newly observed mutations. Additional complications with certain genes, model systems, and assays can create further difficulties with performing timely functional studies. The ACMG/AMP guidelines determine disease-risk levels for each variant involved in Mendelian disease by considering various factors, such as allele frequency, functional analyses, family history, and notably, *in silico* pathogenicity predictions from computational tools [12]. Several computational tools, such as SIFT [17], PolyPhen-2 [18], CADD [19], FATHMM [20], GERP++ [21], PrimateAI [22], REVEL [23], and VEST4 [24], use genomic features to provide pathogenicity scores or categorical predictions for variant interpretation. These tools employ different types of methods, such as functional prediction, evolutionary conservation, and ensemble approaches (Supplementary Table 1). Functional prediction methods assess pathogenicity based on protein or amino acid sequence changes, conservation-based methods use multiple sequence alignments to measure nucleotide site conservation, and ensemble methods often integrate diverse analyses for pathogenicity prediction. Computational predictive tools play a crucial role in assessing the pathogenicity of newly observed variants and can help address some of the challenges posed by conducting functional studies; however, an area of improvement that needs to be addressed is the incorporation of features from protein structures to enhance prediction. Existing tools that use physio-chemical properties include PolyPhen-2 [25], which accounts for changes in accessible surface area, hydrophobic intensity, and B-factor changes that result from nonsynonymous SNPs. Even though these sets of structural features are limited and are often not available for all proteins, they have already resulted in remarkable improvements to PolyPhen-2’s performance. However, an increase in the number of computable quantitative structural features and the number of available protein structures provides more opportunities for additional improvements and allows assessment of protein for which experimental structures are not available. The structure and function of proteins are often intimately tied to the severity or type of disease phenotypes for several Mendelian disorders. Thus, a protein’s structure and function must be considered together to improve the reliability when assessing the impact of genetic variants on disease.

The past few years have yielded significant advancements in protein structure determination due to progress made in structure prediction programs. Meta AI’s ESM Metagenomic Atlas and ESMFold [26], EMBER3D [27], and DeepMind’s AlphaFold2 [28] are a few examples of deep-learning based methods in structure prediction. AlphaFold2 attracted quite a bit of attention at the 14th Critical Assessment of Structure Prediction (CASP14) [29] assessment after predicting the coordinates of backbone atoms of experimentally solved structures within an accuracy of 0.96 Å root mean square deviation (RMSD) and putting forth a different model from their previous entry [30], AlphaFold. AlphaFold2 has generated over 1,068,500 computed structures, complementing the 204,000 experimental structures in the RCSB PDB database [31,32]. This breakthrough enables the study of proteins that lack experimental structures, as obtaining high-resolution protein crystals for structure determination is often challenging.

Despite the critical role of protein structures and the dynamic features that allow these molecular machines to work, there are few pathogenicity prediction programs that consider these spatial and structural features when assessing the impact of a variant on pathogenicity in Mendelian disease. Thus, this study aims to present a case for leveraging protein structures and structure determination programs like AlphaFold2 to improve the classification of missense variants of uncertain significance, provide additional evidence to support imputation of variant pathogenicity, and increase the accuracy of pathogenicity predictions.

In our study, we evaluate different pathogenicity prediction tools and demonstrate a workflow of variant classification by initially using a few sample proteins with no previous experimentally determined structures, namely GALC (galactocerebrosidase) and IRF6 due to their critical roles in rare monogenic disorders. The main protein examined is Interferon Regulatory Factor 6 (IRF6), a transcription factor involved in early development. The IRF family of transcription factors share a highly conserved N-terminal helix-turn-helix DNA-binding domain and a less conserved C-terminal protein-binding domain [33]. Mutations in IRF6 are linked to Van der Woude Syndrome (VWS, OMIM 119300), Popliteal Pterygium Syndrome (PPS, OMIM 119500), and non-syndromic orofacial cleft lip (CL/P, OMIM 119530) [34], [35], [36]]. Despite over 300 known missense variants in IRF6, their impact on protein function and resulting phenotypes remain largely unexplored. Furthermore, there are no liganded or apo experimentally determined protein structures of IRF6 to date. A study by Li et al. conducted a phenotype rescue experiment to assess the functional effects of 37 selected IRF6 variants on orofacial clefts [37]. For each mutation, human variant mRNA was first injected into *irf6*^-/-^ zebrafish at the one-cell stage. Then, the outcomes of the phenotype rescue experiment were categorized as either ‘rescued’ if the variant restored the *irf6* phenotype or ‘ruptured’ if the harmful phenotype persisted. These data provide an experimentally validated set of variants and serve as an excellent example of a protein that benefits from both pathogenicity prediction and structure prediction tools.

## 2 Results

### 2.1 Overview of the goals of the current study

**Fig. 1:**
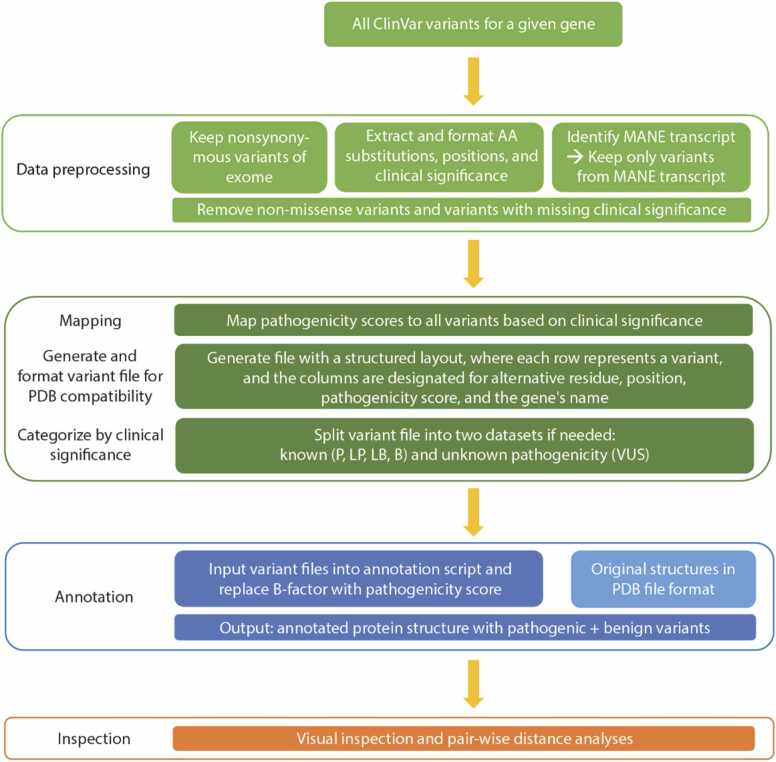
Overview of the workflow for variant annotation of protein structures. All ClinVar variants must first undergo data preprocessing and parsing steps to ensure that only nonsynonymous variants within the relevant transcript that also have data about clinical significance move on to the next steps. The clinical significance categories (P, LP, VUS, LB, B) are assigned a pathogenicity score (see Methods), which is then used to map variants onto the protein structure for further visualization, inspection, and analysis. The other ClinVar class of variants with “conflicting interpretations” is omitted from analysis.

The objectives of this study are to provide a comparative analysis to assess the reliability of various computational tools in predicting the pathogenicity of missense variants and to develop a workflow of VUS classification that leverages insights gained from the final protein structure (Fig. 1). Here, ANNOVAR (see **Methods**) was used to extract precalculated annotations from over 30 pathogenicity prediction tools to generate a collection of pathogenicity predictions for 33 unique missense variants (37 genetic variants in total) in our case study, IRF6. All genetic mutations and their corresponding protein mutations for IRF6 are listed in Table 1. Several amino acid substitutions (G70R, F252L, Q318H) can each result from multiple underlying nucleotide substitutions– these mutations were included in our analysis to further understand the performance of pathogenicity predictors. The results were then used to compare *in silico* pathogenicity predictions to functional assay results and address questions regarding the tool-to-experiment agreement, inter-tool agreement, distribution of computational predictions for each variant, and distribution of predictions for each tool. Then, mutations were mapped onto predicted protein structures from three different prediction programs (AlphaFold2 [28], I-TASSER [38], SWISS-MODEL [39]) to investigate associations between pathogenicity and mutations within the protein or relevant domain.

**Table 1:** All 37 genetic mutations with coordinates and their corresponding 33 protein mutations information of IRF6 (GRCh38/hg38, RefSeq: NM_006147).

Chromosome	Start	End	Reference	Alternative	Amino Acid Change
1	209,788,553	209,788,553	G	A	S424L
1	209,788,556	209,788,556	A	G	I423T
1	209,788,568	209,788,568	A	C	V419G
1	209,788,614	209,788,614	C	T	E404K
1	209,788,625	209,788,625	C	T	R400Q
1	209,788,634	209,788,634	A	G	V397A
1	209,788,638	209,788,638	G	A	P396S
1	209,789,722	209,789,722	A	G	F375S
1	209,790,581	209,790,581	C	T	G325E
1	209,790,601	209,790,601	C	A	Q318H
1	209,790,601	209,790,601	C	G	Q318H
1	209,790,674	209,790,674	A	G	L294P
1	209,790,735	209,790,735	C	T	V274I
1	209,790,783	209,790,783	G	A	P258S
1	209,790,799	209,790,799	G	C	F252L
1	209,790,799	209,790,799	G	T	F252L
1	209,790,801	209,790,801	A	G	F252L
1	209,790,806	209,790,806	C	T	R250Q
1	209,792,271	209,792,271	G	A	P222L
1	209,792,347	209,792,347	G	A	P197S
1	209,796,429	209,796,429	T	C	T100A
1	209,796,465	209,796,465	T	G	N88H
1	209,796,477	209,796,477	G	A	R84C
1	209,796,483	209,796,483	G	T	Q82K
1	209,796,500	209,796,500	G	A	P76L
1	209,796,506	209,796,506	G	A	P74L
1	209,796,519	209,796,519	C	G	G70R
1	209,796,519	209,796,519	C	T	G70R
1	209,796,530	209,796,530	T	G	K66T
1	209,796,549	209,796,549	A	C	W60G
1	209,801,280	209,801,280	C	T	R45Q
1	209,801,281	209,801,281	G	A	R45W
1	209,801,313	209,801,313	T	G	K34T
1	209,801,379	209,801,379	G	A	P12L
1	209,801,388	209,801,388	C	T	R9Q
1	209,801,389	209,801,389	G	A	R9W
1	209,801,398	209,801,398	G	A	R6C

Pathogenicity prediction tools were split into two types: categorical or quantitative tools. Categorical tools classify each variant into discrete groups, such as "deleterious” or "tolerated”. Some categorical tools also provide a quantitative score, but the final prediction often uses thresholds to provide a categorical. Quantitative tools output a numerical score for each variant to describe its likelihood of pathogenicity. Besides AlphaMissense, there are 21 categorical tools and 17 quantitative tools used in this study.

### 2.2 Categorical in silico prediction tools show nontrivial agreement with functional assay results within the protein-binding region

**Fig. 2:**
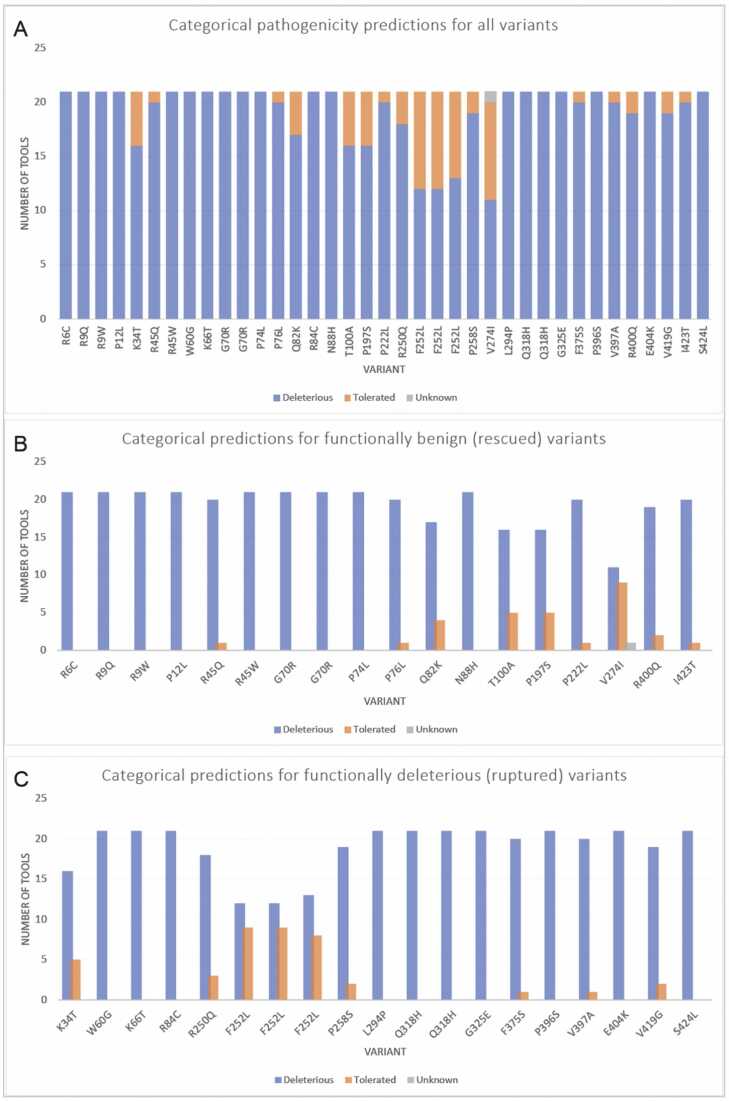
Pathogenicity predictions of IRF6 variants from categorical tools (A) This graph shows the distribution of predictions for each variant from tools that give a categorical prediction (ex. deleterious or benign). Based on these results, computational tools are more likely to predict variants to be deleterious. Interestingly, the nucleotide variants that resulted in an amino acid change in F252 showed varying results, even though all 3 variants result in the same amino acid change. One tool (M-CAP) did not provide a prediction for V274I and is thus marked as unknown. Out of the 37 variants, 19 variants were unanimously predicted to be deleterious by all computational tools. Graphs B and C depict the breakdown of predicted clinical significance from categorical computational tools for (B) variants that ‘rescued’ the irf6 -/- zebrafish (benign) and (C) variants that remained as ‘ruptured’ (deleterious).

Fig. 2 shows the number of times each variant was categorized as deleterious or tolerated by 21 computational tools that provide categorical predictions. The term “deleterious” indicates a higher chance of pathogenicity (Table 2). In Fig. 2A, the variants are ordered from lowest residue position (R6C) to highest position (S424L) on the x-axis. The amino acid residues between P74L and V274I seem more likely to be predicted as “tolerated” than other regions within the protein. One tool (M-CAP) did not provide a prediction for V274I and is thus marked as unknown. There are 14 nucleotide variants within this region – 10 are within the Protein-Binding Domain and the remaining 4 are within the DNA-Binding Domain (Table 1). Notably, 8 of these 14 tolerated variants were found to be functionally benign (rescued), suggesting a nontrivial amount of overlap between the computational predictions and the experimental results in a region with predominantly benign mutations.

**Table 2:** The clarification of equivalent terminologies for harmful and tolerated mutations varies across different pathogenicity prediction tools and the experimental results.

Terminology	Benign Variants	Pathogenic Variants
Experimental Results	Rescued	Ruptured
Pathogenicity Prediction	Benign	Deleterious
	Tolerated	

The functional analysis of IRF6 variants involved assessing their ability to rescue the deleterious irf6 ^-/-^ phenotype in zebrafish through variant mRNA microinjections at the one-cell stage [37]. Fig. 2B and Fig. 2C group computational predictions by whether the nucleotide variant was able to rescue the *irf6*^-/-^ harmful ruptured phenotype in zebrafish [37]. Variants capable or rescuing the harmful ruptured phenotype were then categorized as benign and variants that were incapable of restoring the irf6 variant were categorized as deleterious.

Many benign variants – such as I423T, P222L, P76L, P74L, and G70R – were predominantly predicted to be deleterious. There were also a nontrivial number of tools that predicted functionally harmful variants as benign, as seen in Fig. 2C. Variants V419G, E404K, F375S, and R250Q were all experimentally found to be deleterious, but more than 2 tools predicted these variants to be benign. Although most variants were predicted to be deleterious, it is notable that at least 4 programs accurately predicted some functionally benign variants (R45Q, P12L, R9Q, R9W) as benign or tolerable.

Out of the 37 nucleotide variants, 19 variants were unanimously predicted to be deleterious by all computational tools. Interestingly, the nucleotide variants that resulted in an amino acid change in F252 showed varying results, even though all 3 nucleotide variants result in the same amino acid change. Based on these results, computational tools appear to favor labeling variants as deleterious in the contexts of this gene.

### 2.3 An assessment of inter-tool agreement: quantitative in silico tools show a wide range of scores for pathogenicity predictions

**Fig. 3:**
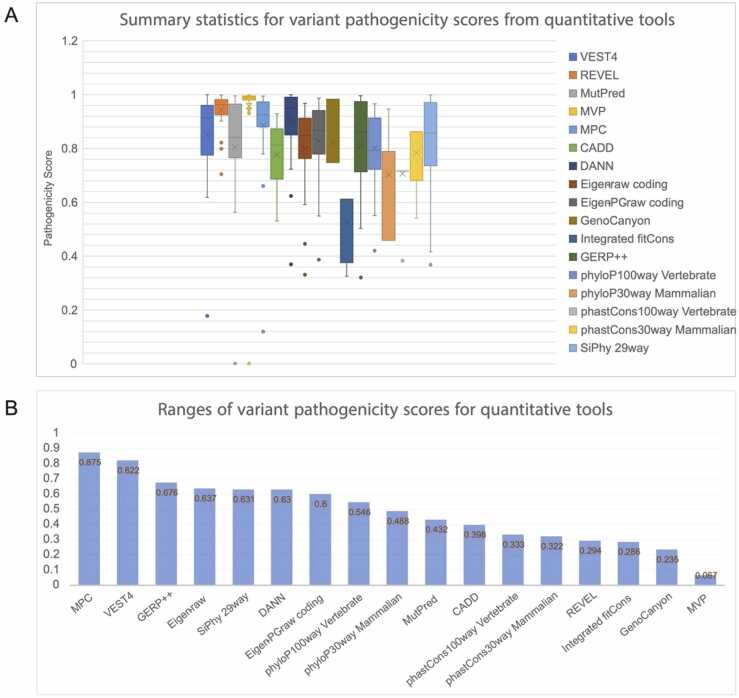
Summary statistics of scores generated by quantitative tools for IRF6 variants (A) Panel A shows summary statistics, such as the median and interquartile range, for the scores generated by each of the quantitative pathogenicity prediction tools for the selected set of IRF6 variants. Each tool outputs a score from 0 to 1, where 1 indicates higher pathogenicity. None of the tools scored any variant as fully benign (0.00) and the data points at 0 correspond to unavailable scores for variant V274I from MVP and from MutPred. (B) Panel B shows the ranges in numerical scores for each tool. MPC and VEST4 have the largest distribution of scores when including outliers, while MVP has the smallest range. Furthermore, MVP scored almost all variants as highly pathogenic.

The second type of tool provides quantitative pathogenicity scores. Fig. 3 shows the range of predictive scores for each of the 17 quantitative tools. Normalized pathogenicity scores range from 0 to 1, with a higher score conferring greater risk of pathogenicity. The range of scores for each program was markedly different. The 3 most conservative tools appear to be REVEL, GenoCanyon, and MVP, since these tools display the smallest ranges in their output scores. The top 6 tools with the most discerning set of scores are MPC, VEST4, DANN, GERP++, Eigen-raw, and SiPhy, since these tools display the largest ranges in their output scores (Fig. 3).

Most of these tools predicted R45W, V274I, and P197S to be the least pathogenic of all given variants (Supplementary Fig. S1) – these 3 variants were found to be benign following functional analysis, thus showing some agreement between functional analysis and computational predictions for benign variants. Notably, some quantitative tools predicted that nucleotide variants C756G and C756A to be more benign than T754C, all of which result in the same amino acid change (F252L). The redundancy of the genetic code poses an interesting challenge to pathogenicity prediction tools based for nonsynonymous variants. Other computational tools, such as MPC and VEST4, scored all 3 nucleotide variants with the same score. Most of these tools, whether quantitative or categorical, tended to skew towards labeling mutants are more likely to be pathogenic than benign.

### 2.4 An assessment of inter-tool agreement amongst categorical tools

We focused on 5 of the 21 categorical tools and assessed the distribution in scores for each variant and within each tool (Table 3). These 5 programs are SIFT, PolyPhen-2, MutationTaster, MutationAssessor, and InterVar. Table 3, which depicts a comparison of 5 tools and their predictions for all 37 variants, is split into 3 categories: 1) inter-tool computational agreement, 2) inter-tool computational uncertainty, and 3) inter-tool computational disagreement. The first 7 variants in Table 3 showed clear agreement amongst the 6 computational tools. There was computational uncertainty in 18 variants, which were generally categorized as VUS and/or of medium pathogenicity. The majority of these 18 variants were found to be deleterious and unable to rescue the *irf6*^*-/-*^ phenotype. The remaining 12 variants showed computational disagreement. Notably, all 12 of these variants rescued the harmful *irf6*^*-/-*^ phenotype, but all the tools predicted these variants to be either pathogenic or of uncertain significance.

**Table 3:** Comparison of predictions from a subset of tools (A) This table shows the computational predictions for each of the 37 variants across 5 categorical tools (SIFT, PolyPhen-2, MutationTaster, MutationAssessor, InterVar) and ClinVar. This table is sorted by computational agreement within these 6 programs – the last three columns quantify the inter-tool agreement. The variant with the greatest amount of inter-tool computational agreement is at the top and the variants with no computational agreement are listed at the bottom. A “Yes” signifies agreement between the functional and predicted results, “No” signifies disagreement, and “VUS” indicates that the predictive tool did not provide a binary answer, thus a comparison cannot be made. Since ClinVar is a user-dependent database, not all variants are recorded – “N/A” indicates that this variant was not found in ClinVar. Notably, R84C was unanimously predicted to be pathogenic by all 5 computational tools. At the bottom, there are 5 rows that provide a summary for each tool. The row labeled “Total # of Agreements” shows the number of variants that had agreement between the functional assay results and the computational tool in that column.

Domain	Variant	Functional Results	SIFT	PolyPhen2	MutationTaster	MutationAssessor	InterVar	ClinVar	# Agreements	# Disagreements	# All Else
**Computational Agreement**											
DNA-binding	R84C	Ruptured	Yes	Yes	Yes	Yes	Yes	Yes	6	0	0
C-terminal	S424L	Ruptured	Yes	Yes	Yes	VUS	Yes	Yes	5	0	1
C-terminal	P396S	Ruptured	Yes	Yes	Yes	VUS	Yes	Yes	5	0	1
DNA-binding	W60G	Ruptured	Yes	Yes	Yes	Yes	VUS	N/A	4	0	2
Protein-Binding	V274I	Rescued	No	No	Yes	Yes	Yes	Yes	4	2	0
Protein-Binding	R250Q	Ruptured	Yes	Yes	Yes	No	VUS	Yes	4	1	1
C-terminal	E404K	Ruptured	Yes	Yes	Yes	VUS	VUS	Yes	4	0	2
**Computational Uncertainty**											
DNA-binding	K66T	Ruptured	Yes	Yes	Yes	VUS	VUS	N/A	3	0	3
Protein-Binding	F375S	Ruptured	Yes	Yes	Yes	VUS	VUS	N/A	3	0	3
Protein-Binding	G325E	Ruptured	Yes	Yes	Yes	VUS	VUS	N/A	3	0	3
Protein-Binding	Q318H	Ruptured	Yes	Yes	Yes	VUS	VUS	N/A	3	0	3
Protein-Binding	Q318H	Ruptured	Yes	Yes	Yes	VUS	VUS	N/A	3	0	3
Protein-Binding	L294P	Ruptured	Yes	Yes	Yes	VUS	VUS	N/A	3	0	3
Protein-Binding	P258S	Ruptured	Yes	Yes	Yes	No	VUS	N/A	3	1	2
C-terminal	V419G	Ruptured	Yes	Yes	Yes	VUS	VUS	N/A	3	0	3
C-terminal	V397A	Ruptured	Yes	Yes	Yes	No	VUS	N/A	3	1	2
DNA-binding	T100A	Rescued	Yes	No	No	Yes	VUS	N/A	2	2	2
DNA-binding	Q82K	Rescued	Yes	No	No	Yes	VUS	N/A	2	2	2
DNA-binding	K34T	Ruptured	No	Yes	Yes	No	VUS	N/A	2	2	2
Mid-Linker	P197S	Rescued	No	Yes	No	Yes	VUS	N/A	2	2	2
Mid-Linker	P222L	Rescued	No	No	No	Yes	VUS	VUS	1	3	2
Protein-Binding	F252L	Ruptured	No	No	Yes	No	VUS	N/A	1	3	2
Protein-Binding	F252L	Ruptured	No	No	Yes	No	VUS	N/A	1	3	2
Protein-Binding	F252L	Ruptured	No	No	Yes	No	VUS	N/A	1	3	2
C-terminal	R400Q	Rescued	Yes	No	No	VUS	No	No	1	4	1
**Computational Diagreement**											
N-terminal	P12L	Rescued	No	No	No	VUS	VUS	N/A	0	3	3
N-terminal	R9Q	Rescued	No	No	No	VUS	No	No	0	5	1
N-terminal	R9W	Rescued	No	No	No	No	No	No	0	6	0
N-terminal	R6C	Rescued	No	No	No	VUS	No	No	0	5	1
DNA-binding	N88H	Rescued	No	No	No	VUS	VUS	No	0	4	2
DNA-binding	P76L	Rescued	No	No	No	VUS	VUS	N/A	0	3	3
DNA-binding	P74L	Rescued	No	No	No	VUS	VUS	N/A	0	3	3
DNA-binding	G70R	Rescued	No	No	No	VUS	VUS	N/A	0	3	3
DNA-binding	G70R	Rescued	No	No	No	VUS	VUS	N/A	0	3	3
DNA-binding	R45Q	Rescued	No	No	No	VUS	VUS	VUS	0	3	3
DNA-binding	R45W	Rescued	No	No	No	No	VUS	N/A	0	4	2
C-terminal	I423T	Rescued	No	No	No	VUS	VUS	N/A	0	3	3

Summary	SIFT	PolyPhen2	MutationTaster	MutationAssessor	InterVar	ClinVar
**Total # of Agreements**	18	17	20	7	4	6
**Total # of Disagreements**	19	20	17	9	4	5
**Total # of Uncertainties**	0	0	0	21	29	2
**Total # of N/A**	0	0	0	0	0	24
**Total # Variants**	37	37	37	37	37	37

Supplementary Fig. S2 illustrates the distribution of predictions made by each tool, helping us assess differences in the likelihood of a tool classifying a variant as pathogenic. This provides further evidence that these tools may be more reluctant to categorize a variant as benign, thus leading to greater inaccuracy when it comes to tolerated mutants.

Each tool has a different system of categorization. For example, SIFT has only two potential categories: deleterious or tolerated. Other tools, such as MutationAssessor and ClinVar, provide more intermediate categories. These middle categories provide room for deeming certain variants as variants of uncertain significance. This begs the question: does a greater number of categories denote greater alignment with the functional assay’s results? MutationAssessor deemed 4 variants as highly pathogenic (R84C, W60G, R9W, R45W) but two of these were able to rescue the *irf6-/-* phenotype, while the other two did not.

**Fig. 4:**
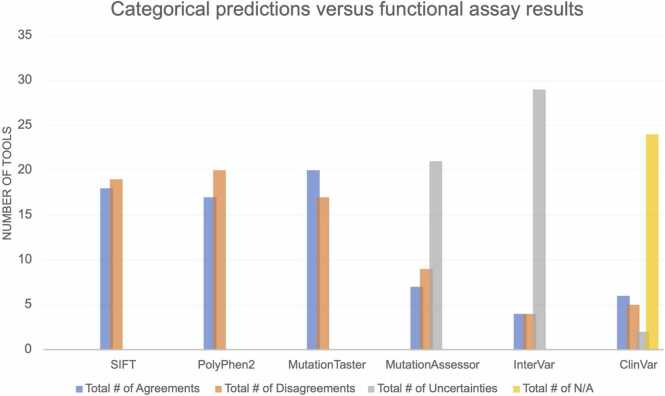
Pathogenicity result comparison between Functional Assay and 6 Tools for IRF6 variants. For each of these categorical tools, approximately 50% of the variants have discordant results between experimental results and computational predictions. Notably, even ClinVar annotations show a discrepancy with experimental results, although many variants were not present in ClinVar.

On the other hand, MutationTaster categorized these variants into one of 3 groups – Disease_causing_automatic, Disease_causing, or Polymorphism_automatic. MutationTaster displayed the greatest alignment with the functional assay: the predictions for 20 out of 37 variants were in alignment with the functional assay (Table 3). Even though some tools have a greater range of categories, this does not definitively denote greater alignment with functional results. Lastly, Fig. 4 shows the agreement between these 5 categorical tools and the results of the functional assay. MutationTaster, SIFT, and PolyPhen-2 have the greatest number of agreements with the functional assay results. MutationAssessor and InterVar categorized most variants as VUS or of medium pathogenicity.

Apart from the 33 variants that have been experimentally validated, we identified an additional 70 variants considered potentially benign obtained from the newly released gnomAD v4.0 [40] database. We filtered for variants with a minor allele frequency (MAF) exceeding 5%, classifying them as potentially benign. The majority (66) of these variants were located in untranslated regions or introns. Among the remaining 4 coding variants, 3 were synonymous, and one was non-synonymous (V274I). Notably, the non-synonymous variant V274I was already included in the original set of experimentally validated variants. We also extended our search to the gnomAD v4.0 exome database for variants occurring at a MAF> 5 × 10^-5^ and identified one new non-synonymous variant, R400P, with a high predicted likelihood of pathogenicity from most of the categorical variant effect predictors.

### 2.5 Insights into pathogenicity from protein structure

Given some of the shortcomings of current pathogenicity prediction tools, we explored the idea of integrating protein structures to improve the interpretation of these predictions. We plotted these 37 mutations on predicted protein structures of IRF6 to investigate any correlations between pathogenicity and the location of the mutation within the protein or a given domain. Furthermore, we can glean information about the significance of each mutation based on its location and associated functional result.

**Fig. 5:**
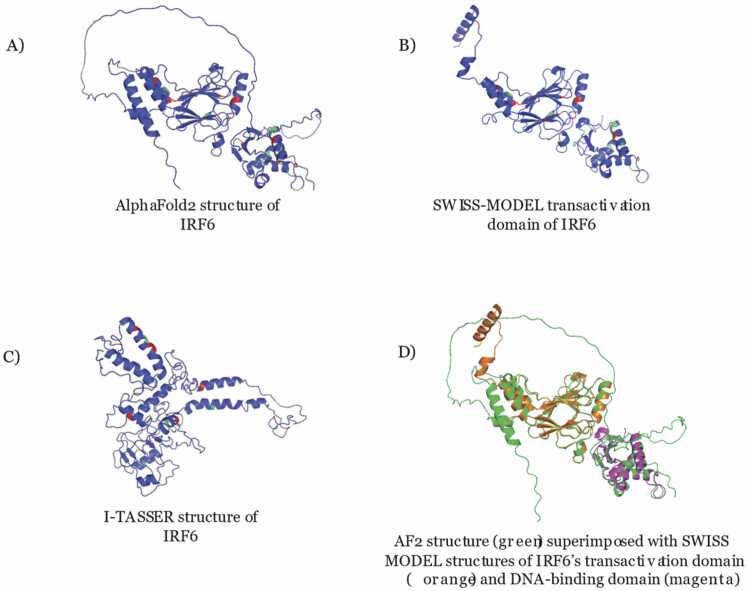
Predicted Protein Structures from different programs These images show (A) the AlphaFold2 structure prediction of IRF6 (residues 1–467), (B) the SWISS-MODEL structure prediction of the transactivation domain of IRF6 (residues 214–445), and (C) the I-TASSER structure prediction of IRF6 (residues 1–467) of highest confidence. Green residues were benign and red residues were pathogenic according to functional analysis. (D) The two SWISS-MODEL structures of the IRF6 transactivation and DNA-binding domains (residues 9–122) were superimposed onto the AlphaFold2 predicted structure.

**Fig. 6:**
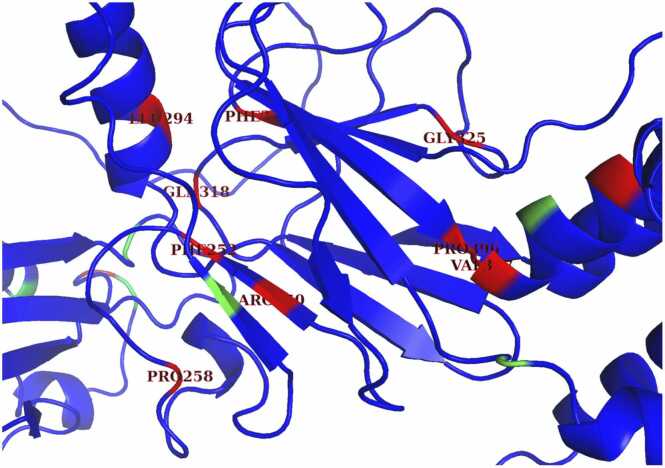
Potential visible binding pocket in protein-binding domain. This image shows a zoomed-in version of the AlphaFold2 structure of full IRF6. Some of the residues in or near the beta sheet, which is mainly part of the protein-binding domain, were categorized as ruptured. Although some of these residues are distant from each other in the amino acid sequence, there are some pairs that are close together in the 3D folded structure, such as ARG-250 and PHE-375. The clustering of red mutations in the protein-binding domain highlights the significance of this domain for IRF6 protein function and provide insight on which proteins may be more likely to be deleterious.

At the time of this study, there is no experimentally determined structure of liganded or apo IRF6. Three predicted protein structures were generated from AlphaFold2 [28], SWISS-MODEL [39], and I-TASSER [38] (Fig. 5). AlphaFold2 and I-TASSER show full-length protein structures, while SWISS-MODEL provides two separate structures of the transactivation domain (residues 214–445) and DNA-binding domain (residue 9–122). Green residues rescued the harmful ruptured *irf6-/-* phenotype, while red residues did not alter the ruptured phenotype. Although these three structures look visibly quite different, one important aspect of the protein-binding domain – the beta sheet – is conserved in both the AlphaFold2 structure and SWISS-MODEL structures. Several of the mutations that were categorized as deleterious were clustered within or around the protein binding domain, specifically the beta sheets. This spatial cluster further underlines the significance of the protein-binding domain and the impact of a nucleotide variant on protein function (Fig. 6). The clustering of red mutations in the protein-binding domain highlights the significance of this domain for IRF6 protein function and provides insight into which variants are more likely to be deleterious. Interestingly, none of the 4 N-terminal variants (R6C, R9Q, R9W, P12L) were categorized as ruptured (harmful), but 3 of the 4 were categorized as deleterious by the subset of 5 categorical tools.

Clusters of pathogenic mutations can be visualized via pyMOL or other molecular visualization software that handle PDB files, such as VMD (see Methods). Quantitative measures such as distance analyses can be further applied to understand the presence of pathogenic clusters and their biological relevance. Given that structure informs most proteins’ functions and vice versa, there are certainly vital regions of the protein that cannot tolerate variants or can only tolerate a limited amount of variation. Understanding the pattern behind mutational clusters may provide additional evidence that can help us classify variants of uncertain significance in a timelier manner.

**Fig. 7:**
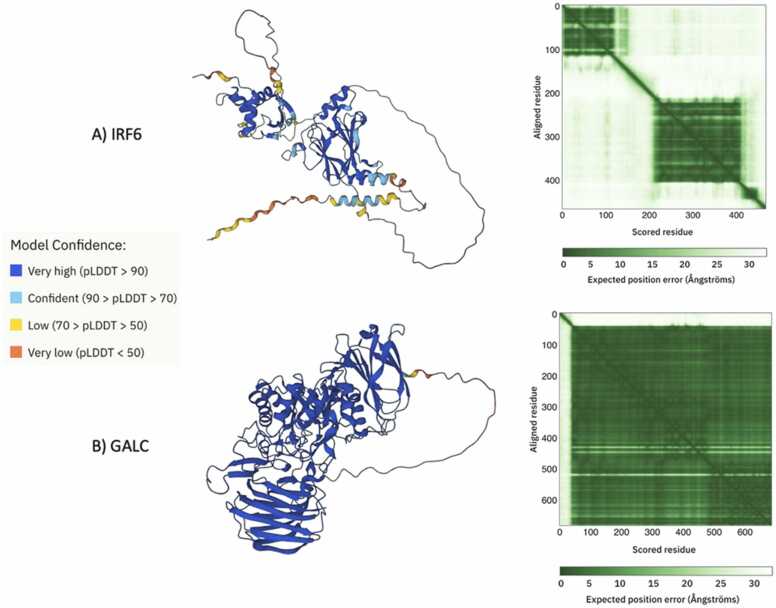
A) Image of the AlphaFold2 prediction for IRF6, color coded by confidence score and the Predicted Aligned Error (PAE) from AlphaFold2 database. The color at position (x, y) indicates AlphaFold2’s expected position error at residue x, when the predicted and true structures are aligned on residue y. The PAE plot is useful for assessing the inter-domain accuracy of the predicted structure. Dark green indicates low error and light green indicates high error. Each of the squares corresponds to a domain. (B) Image of the AlphaFold2 prediction for GALC, color coded by confidence score and the Predicted Aligned Error (PAE) from AlphaFold2 database. These images were sourced directly from AlphaFold2 and the legend was adapted from AlphaFold2.

However, it must be addressed that not all proteins will exhibit a pattern of mutational clustering. Another protein implicated in the cause of rare diseases is galactocerebrocidase (GALC or Galactosylceramidase), which is a lysosomal enzyme that hydrolyzes galactose ester bonds of galactosylceramide, galactosylsphingosine, and other molecules that are important to produce myelin. Mutations in GALC are typically associated with Krabbe Disease (OMIM 245200), a rare autosomal recessive disorder characterized by absence of myelin, severe gliosis, and other neurodegenerative symptoms resulting from the accumulation of toxic myelin breakdown product. There are over 300 known mutations for GALC in ClinVar alone, and over 200 of these variants are VUS. GALC has no known full, human, experimentally determined protein structure as of now in the RCSB PDB database. Applying this method of visualization to other proteins brought some cases where distance analysis is not as effective, as seen in GALC. When known mutations are plotted on a predicted structure of GALC, clusters are not apparent, and variants are seemingly distributed across all motifs (Figs. 7, S3). Such examples are also fruitful, as these patterns could shed light on a protein’s overall mutational tolerance.

### 2.6 Analysis on AlphaMissense

Recently, Cheng et al. developed AlphaMissense [41], which is a deep learning model that aims to build on structure predictions from AlphaFold2 to help classify all amino acid substitutions. AlphaMissense classifies 32% of all missense variants in the human genome as likely pathogenic and 57% as likely benign using a cutoff yielding 90% precision on the ClinVar dataset. Intrigued by this new development, we compared the pathogenicity predictions from AlphaMissense for each of the relevant IRF6 variants to the functional testing results (Supplementary Fig. S4). AlphaMissense predicted 34 of all 37 variants to be pathogenic, resulting in a high false positive rate (FPR) of 83.3% (15/18 variants). Removing the 4 redundant missense variants (G70R, two F252L, Q318H) results in a similarly high FPR of 82.3% (14/17 variants). While 3 genetic variants (P197S, V274I, R400Q) that were predicted as benign by AlphaMissense were indeed found to rescue the abnormal phenotype in the *irf6*^*-/-*^ zebrafish model, the remaining 15 benign variants were misclassified as pathogenic, contradicting the functional results, and suggesting subpar performance for this particular gene.

## 3 Discussion

A key challenge in human genetics is determining how nucleotide variation will impact protein function and potential disease-casual effect [11]. Thus, the objective of this paper was to funnel new insights from structural biology and protein structures into available genetic data to determine how we can improve the variant classification challenge. Here, we aimed to assess the current state of pathogenicity prediction tools and provide a case to leverage protein structure determination tools, like AlphaFold2, and genetic variant databases to improve the re-classification of variants of uncertain significance. The number of variants of uncertain significance will only continue to rise. Therefore, we need to develop sustainable methods and frameworks that can help assess the clinical significance of newly observed variants in a timely and accurate fashion. Furthermore, the continuing increase in variant data will only further improve the robustness of these methods. Here, we hoped to demonstrate that structural information can facilitate the re-interpretation of variants of unknown significance and that predicted structures can help accelerate the process. Since these structures and ideas can now be widely applied to almost all proteins in the human genome, the combination of structure prediction and structure-based variant scoring will facilitate the variant interpretation.

Here, we used the protein IRF6 as a use case for leveraging protein structure prediction programs and as a case study to determine the reliability of different computational tools in predicting the pathogenicity of a set of exonic, nonsynonymous variants in the protein IRF6. To achieve this, ANNOVAR [42] was ran on 37 IRF6 mutations using over 30 computational tools – the output is a set of pathogenicity predictions for each variant from each of the computational tools. We found that computational tools tend to skew towards categorizing variants as pathogenic rather than benign. Of the 37 variants, 19 were unanimously declared as deleterious by all 21 tools that provided a categorical prediction. However, certain regions of the protein were more likely to be predicted as benign than other regions. Residues between P74L and V274I were more often predicted as tolerated than other regions within the protein. Of the 14 nucleotide variants within this region, 8 were also found to be benign according to the phenotype rescue experiments (Table 3), suggesting a nontrivial amount of overlap between the computational predictions and the experimental result in a region with predominantly benign mutations.

There are a few different types of variant databases available to researchers such as population frequency, disease-specific, or variant functional prediction databases. The Genome Aggregation Database (gnomAD) [43], which contains data from the Exome Aggregation Consortium (ExAc) [44], and the 1000 Genomes Project [45] are examples of databases used to obtain the frequencies of variants in large populations. Disease databases that contain pathogenic variants and known associated information or annotations include NIH ClinVar [46], OMIM [47], Human Gene Mutation Database [48], and DECIPHER [49]. These public and private repositories aid researchers and clinicians in cataloging known variants and their associated clinical significance and can serve as a useful tool to inform variant classification. Here, we compare our computational and experimental results to entries from NIH ClinVar. It must also be noted that ClinVar is a public database that takes user submissions, and so ClinVar provides information for a subset of observed variants. Furthermore, pathogenic variants are perhaps more likely to be observed and recorded than benign variants, thus skewing the database towards accounting for more pathogenic variants.

Different tools have different methods of determining pathogenicity level and provide different kinds of outputs. When it comes to tools that provide purely quantitative results, programs that range from conservative to more discerning. Predictive tools with intermediate categories between deleterious and benign tend to categorize more mutations as variants of uncertain significance or of medium pathogenicity. In this analysis, MutationTaster displayed the greatest agreement between the predictions and the experimental results since the predictions for 20 out of 37 variants were in alignment with the functional assay.

Some examples of features that may improve pathogenicity prediction are structural features that incorporate spatial information from the protein (Fig. 6). This helps incorporate more a priori biochemical knowledge to determine how the function of the protein may be influenced by altered biochemistry and biophysics. Some examples of basic features are the variant’s position along the amino acid sequence, mutational tolerance, and mutational frequency within a domain since these features could help us better predict the existence of pathogenic mutations in that domain or spatial neighborhood. Conducting clustering analyses can also help find binding pockets that be important for binding and protein function that may affecting pathogenicity.

Another area of improvement for predictive tools is considering the biological role of protein domains and incorporating gene-specific features. In this analysis, the 4 N-terminal variants were experimentally shown to rescue the harmful ruptured phenotype [37], but 3 of these 4 were categorized as deleterious by the subset of categorical tools. The N-terminal domain typically plays crucial roles in protein assembly and folding, whereas the C-terminal domain is typically important for substrate protein binding and stabilization. The categorization of N-terminal variants as deleterious might be influenced by the fact that the N-terminal DNA-binding domain is highly conserved in IRF protein family [33]. However, if certain N-terminal mutations are tolerable and do still allow the protein to properly fold, they may be less likely to have a downstream pathogenic effect since these residues may not play as large a role in substrate binding and catalysis. Changes in hydrophobicity, distances from other pathogenic or benign mutations, the presence or disruption of disulfide bridges, disordered binding regions, surface area, and changes in free energy are just a few examples of quantitative, gene-specific features that can be incorporated into pathogenicity prediction tools to improve their predictive power.

The variance in structure predictions across different tools can also complicate the selection of the best structure for downstream analysis. This decision may also be facilitated by our variant analysis since we want to assess where mutations are mapped to the structures and then assess how benign clusters separate from pathogenic cluster. The variance in predictions can partially be addressed by comparing the predicted structure of a protein of interest to its homologs or other closely related proteins with existing full or partial structures.

The protein structure-based evaluation of missense variants are generally divided into the algorithms that calculate the difference in free energy (ΔΔG) and those that use structural features without ΔΔG [50]. When it comes to protein structure prediction, AlphaFold2 and other prediction programs have revolutionized the field of structural biology and provided a powerful tool for accelerating biomedical research. IRF6 currently has no experimentally determined structure to our knowledge, so this presented a clinically relevant opportunity to leverage AlphaFold2 and generate a predicted structure for structurally informed pathogenicity analysis. A similar study by McDonald et. al assessed AlphaMissense pathogenicity predictions of missense variants in cystic fibrosis transmembrane conductance regulator (CFTR), which is heavily implicated in the development of another monogenic disease, cystic fibrosis (CF) [51]. Their analyses suggest that although AlphaMissense performed adequately on predicting the pathogenicity of severe CF mutations, AlphaMissense similarly yielded a high false positive rate on a set of common CFTR missense variants. While AlphaMissense has good overall performance genome-wide, its performance may vary greatly between different genes since a general-purpose model for all human genes was built by AlphaMissense. The fact that AlphaMissense is built on AlphaFold2 also raises concern as AlphaFold2 tends to have worse performance predicting 3D protein structure of missense mutations compared to wild-type structures [52]. In summary, these results highlight that while AlphaMissense has provided a valuable resource for functional interpretation of genetic variants in this field, there is still considerable room for improvement in increasing the accuracy of these computational tools. Worth mentioning, there are other similar modern based transformer predictors for deleterious variants, including MutFormer [53] and ESM1b [54]. This further underscores the importance of supplementing general-purpose models with specialized, gene-centric studies, data, and models to better contribute to our understanding of genetic variant classification and associated challenges.

Moreover, experimental validation and variant-related phenotype research are still in need, which could provide great advantage in verifying and improving computational prediction. Patient phenotype information related to IRF6, such as hypodontia [55], could be incorporated in the tools to improve computational prediction. Combing the wealth of data generated by next generation sequencing with the groundbreaking advancements in structural biology presents a ripe opportunity to improve pathogenicity prediction. Furthermore, the importance of incorporating specialized, gene-specific data into the process of variant classification cannot be overlooked. This study represents an exploratory analysis on using predicted structures to gain novel biological insights into the impact of the missense variants on pathogenicity in a clinically relevant gene.

## 4 Methods

### 4.1 Bioinformatics methods

A master file containing several thousand structural nucleotide variants was sourced from ANNOVAR [42]. The file was then filtered for IRF6 missense mutations that were nonsynonymous. Next, the 33 missense mutations from Li et al.’s functional study were selected. Only mutations found on NM_006147.4, which corresponds to the IRF6 transcript used in the functional assays, moved on to the next step. This selection process resulted in 37 unique IRF6 structural variants. There are four additional variants to the original 33 since some resulted in the same functional amino acid change but were caused by different nucleotide variants. Next, a separate file (.avinput extension) was created that contained the following 5 pieces of information formatted for ANNOVAR input: chr, start, end, reference allele, and alternative allele(s). It was ensured that all relevant ANNOVAR databases were up to date and were compatible with hg38. The main database is dbNSFP42a, which is a whole-exome dataset that contains several transcript-specific functional predictions and annotations for human nonsynonymous SNVs from various predictive tools. Besides AlphaMissense [41], all other predictive tools used in this study are summarized and cited in the Supplementary Table 1. In addition to the main databases, gnomad_exome and clinvar_20221231 were also downloaded. Lastly, to obtain functional prediction of variants in whole-exome data, the prepared avinput file was ran through ANNOVAR with dbNSFP42a using a command that runs table_annovar.py using GRCh38 and 7 databases, then outputs a csv file with numerical prediction scores, normalized scores, and/or a categorical prediction from each tool for each variant.

### 4.2 Protein structure methods

The protein structures were obtained as PDB files from 3 sources. The AlphaFold2 (AF2) structure is a full-length, monomeric, unliganded structure for human IRF6 and was directly downloaded from the AF2 database. The amino acid sequence used in AF2 is sourced from UniProt and the length of the IRF6 sequence is 467aa. It must be acknowledged that the model confidence for residues 116–218 is low or very low (pLDDT < 70), but the residues comprising the protein-binding domain have very high confidence (pLDDT >90). The SWISS-MODEL transactivation domain and DNA-binding domain for IRF6 were readily available and downloaded. The I-TASSER structure was generated using a FASTA file for full-length IRF6 from NIH NCBI. The I-TASSER output contained 5 predicted structures – the model with the highest overall confidence was chosen for further analysis. To measure the similarity between any two superimposed structures, the RMSD for each pair of proteins was calculated using pyMOL. Five aligned pairs of structures were considered: AF2—SWISS-MODEL transactivation domain, AF2—SWISS-MODEL DNA-binding domain, AF2—I-TASSER, I-TASSER—SWISS-MODEL transactivation domain, and I-TASSER—SWISS-MODEL DNA-binding domain. The resulting RMSD values for these alignments were 0.74 Å, 0.69 Å, 31.31 Å, 27.33 Å, and 16.81 Å respectively. These values indicate that structures from AF2 and SWISS-MODEL are most similar to each other and the structure from I-TASSER is quite different from the other predicted structures.

To efficiently map variants and their corresponding functional result (‘rescued’ or ‘ruptured’) onto the protein structure, numerical scores were assigned to each result and inputted into the ‘B-factor’ column of the PDB file such that rescued and ruptured variants were distinctly marked on the visualization. A separate plain text file was created that contains the amino acid change, residue code, position, and pathogenicity score for each nucleotide variant of interest. This file served as the input for a script that outputs a modified PDB file annotated with the pathogenicity score at each relevant variant and can be visualized on any molecular visualization software, such as pyMOL.

The mapping process for GALC is similar to the steps above. First, the AF2 structure is a full-length, monomeric structure with a sequence length of 685aa and high confidence (pLDDT >90) for all residues after 1–40. The first 40 residues comprise the signal peptide at the N-terminus. Then, all missense variants with a corresponding clinical significance were downloaded from ClinVar. Variants with “conflicting interpretations” of clinical significance in ClinVar were omitted from analysis. Since AF2 draws its amino acid sequence from UniProt, the UniProt FASTA was aligned with the MANE transcript from ClinVar for each protein to ensure a consensus. Only variants from the MANE transcript were later processed and mapped onto their respective AF2 predicted structures.

## Declaration of Competing Interest

The authors have no conflicts of interest to declare.
