# Supplementary material for: Genetic variant classification by predicted protein structure: A case study on IRF6
Source: Comput Struct Biotechnol J. 2024 Feb 3;23:892–904. doi: 10.1016/j.csbj.2024.01.019 (PMC10869248; doi:10.1016/j.csbj.2024.01.019)
Supplement: Supplementary Table 1 and Figures — Supplementary material [file mmc1.docx]

**Supplementary Table 1. Existing *in silico* pathogenicity prediction tools, their main modes of operation, and the main outputs used in this study. Different settings with separate prediction result for the same tool were further clarified as *italic* after the tool.**

| **Tool** | **Mode of Operation** | **Output used** |
| --- | --- | --- |
| PolyPhen-2[1]  *(PolyPhen-2 HDIV, PolyPhen-2 HVAR)* | Function prediction | Categorical |
| SIFT[2] | Function prediction | Categorical |
| SIFT4G[3] | Function prediction | Categorical |
| FATHMM[4]  *(FATHMM, FATHMM-XF)* | Function prediction | Categorical |
| integrated fitCons[5] | Function prediction/ Conservation | Quantitative |
| LRT[6] | Function prediction | Categorical |
| MutationAssessor[7] | Function prediction | Categorical |
| MutationTaster[8] | Function prediction | Categorical |
| VEST4[9] | Function prediction | Quantitative |
| MutPred[10] | Function prediction | Quantitative |
| DEOGEN2[11] | Function Prediction | Categorical |
| PROVEAN[12] | Function prediction/ Conservation | Categorical |
| GERP++[13] | Conservation | Quantitative |
| phastCons[14]  *(phastCons100way vertebrate, phastCons30way mammalian)* | Conservation | Quantitative |
| phyloP[15]  *(phyloP100way vertebrate, phyloP30way mammalian)* | Conservation | Quantitative |
| SiPhy 29way[16] | Conservation | Quantitative |
| LIST-S2[17] | Conservation | Categorical |
| Meta-SVM[18] | Conservation | Categorical |
| MVP[19] | Conservation | Quantitative |
| M-CAP[20] | Conservation | Categorical |
| Eigen[21]  *(Eigen-raw coding, Eigen-PC-raw coding)* | Conservation | Quantitative |
| GenoCanyon[22] | Conservation/ Biochemical | Quantitative |
| FATHMM-MKL[23] | Conservation | Categorical |
| FATHMM-XF[4] | Conservation | Categorical |
| MetaRNN[24] | Ensemble | Categorical |
| PrimateAI[25] | Ensemble | Categorical |
| CADD[26] | Ensemble | Quantitative |
| DANN[27] | Ensemble | Quantitative |
| MPC[28] | Ensemble | Quantitative |
| REVEL[29] | Ensemble | Quantitative |
| MetaLR[30] | Ensemble | Categorical |
| ClinPred[31] | Ensemble | Categorical |
| BayesDel[32]  (add AF and noAF) | Ensemble | Categorical |

**
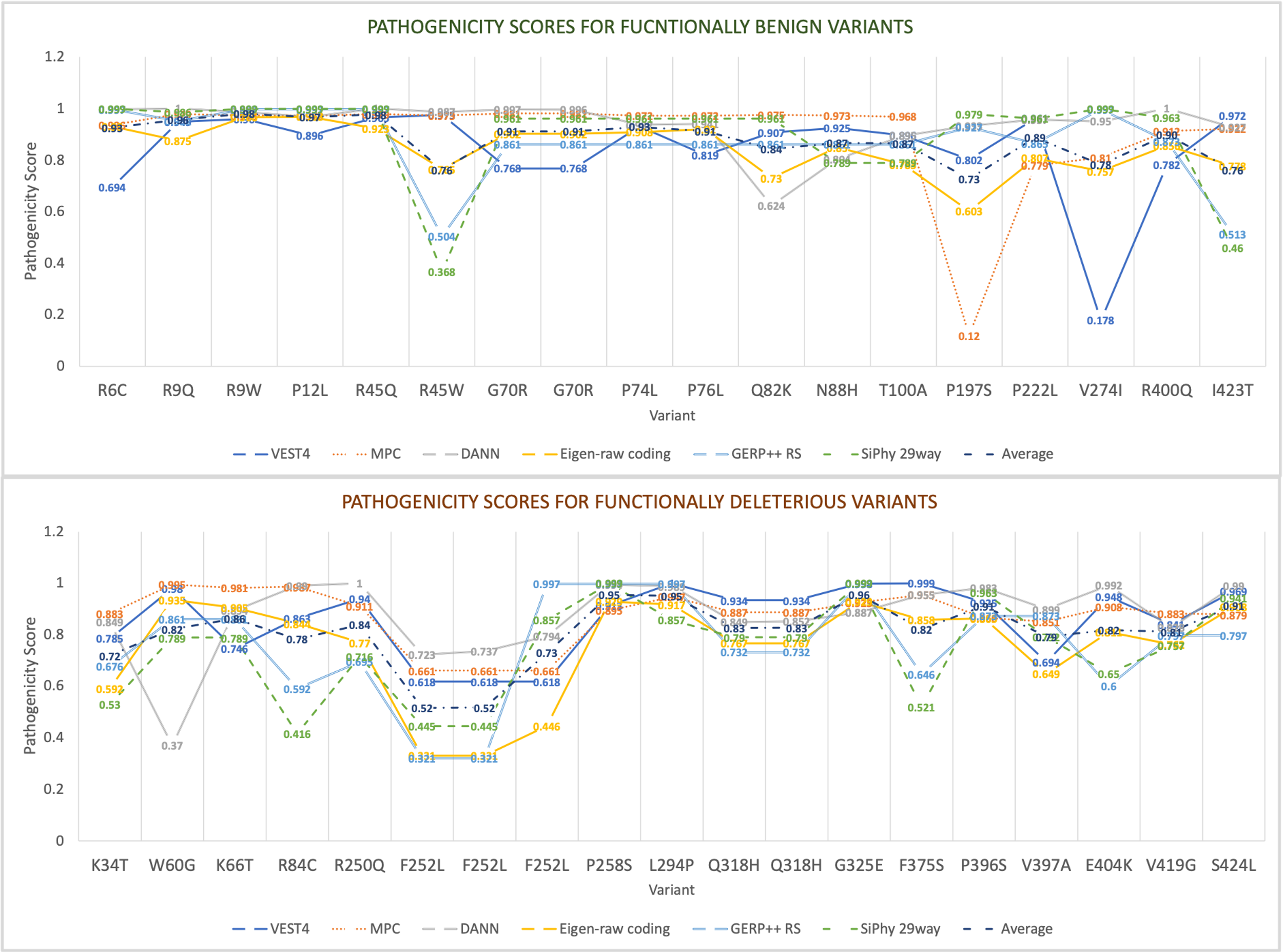
**

**Figure S1. Pathogenicity scores predicted by top 6 most discerned tools in terms of benign variants and deleterious variants based on experimental results.**

**Figure S2. Distribution of predictions for different categories made by various tools.**

**
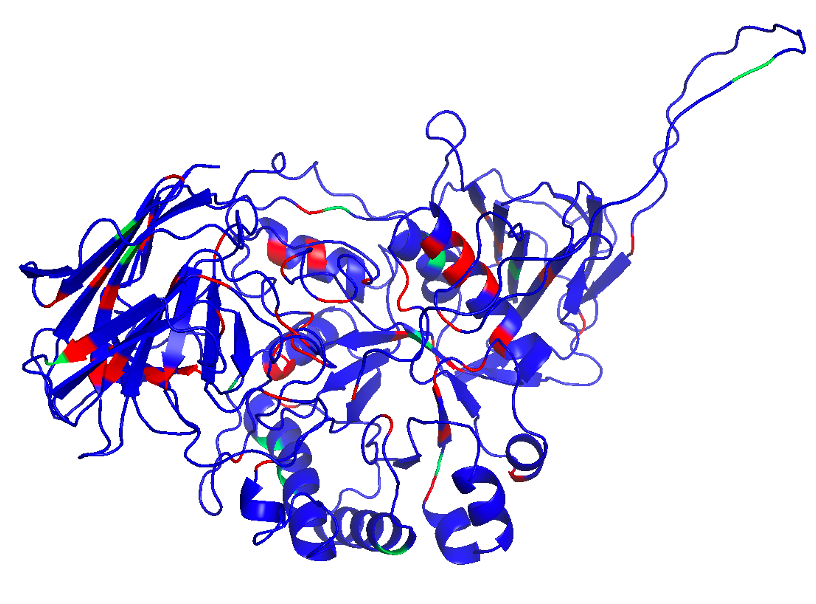
**

**Figure S3. AlphaFold2 structure of GALC with known mutations from ClinVar.**

**Figure S4. Pathogenicity predictions from AlphaMissense for each and the functional results of the relevant IRF6 variants.**

**Reference:**

[1] I. Adzhubei, D. M. Jordan, and S. R. Sunyaev, “Predicting functional effect of human missense mutations using PolyPhen-2,” *Curr. Protoc. Hum. Genet.*, vol. Chapter 7, p. Unit7.20, Jan. 2013, doi: 10.1002/0471142905.hg0720s76.

[2] P. C. Ng and S. Henikoff, “SIFT: Predicting amino acid changes that affect protein function,” *Nucleic Acids Res.*, vol. 31, no. 13, pp. 3812–3814, Jul. 2003, doi: 10.1093/nar/gkg509.

[3] R. Vaser, S. Adusumalli, S. N. Leng, M. Sikic, and P. C. Ng, “SIFT missense predictions for genomes,” *Nat. Protoc.*, vol. 11, no. 1, Art. no. 1, Jan. 2016, doi: 10.1038/nprot.2015.123.

[4] M. F. Rogers, H. A. Shihab, M. Mort, D. N. Cooper, T. R. Gaunt, and C. Campbell, “FATHMM-XF: accurate prediction of pathogenic point mutations via extended features,” *Bioinforma. Oxf. Engl.*, vol. 34, no. 3, pp. 511–513, Feb. 2018, doi: 10.1093/bioinformatics/btx536.

[5] B. Gulko, M. J. Hubisz, I. Gronau, and A. Siepel, “Probabilities of Fitness Consequences for Point Mutations Across the Human Genome,” *Nat. Genet.*, vol. 47, no. 3, pp. 276–283, Mar. 2015, doi: 10.1038/ng.3196.

[6] S. Chun and J. C. Fay, “Identification of deleterious mutations within three human genomes,” *Genome Res.*, vol. 19, no. 9, pp. 1553–1561, Sep. 2009, doi: 10.1101/gr.092619.109.

[7] B. Reva, Y. Antipin, and C. Sander, “Predicting the functional impact of protein mutations: application to cancer genomics,” *Nucleic Acids Res.*, vol. 39, no. 17, p. e118, Sep. 2011, doi: 10.1093/nar/gkr407.

[8] R. Steinhaus, S. Proft, M. Schuelke, D. N. Cooper, J. M. Schwarz, and D. Seelow, “MutationTaster2021,” *Nucleic Acids Res.*, vol. 49, no. W1, p. W446, Jul. 2021, doi: 10.1093/nar/gkab266.

[9] H. Carter, C. Douville, P. D. Stenson, D. N. Cooper, and R. Karchin, “Identifying Mendelian disease genes with the Variant Effect Scoring Tool,” *BMC Genomics*, vol. 14, no. Suppl 3, p. S3, May 2013, doi: 10.1186/1471-2164-14-S3-S3.

[10] V. Pejaver *et al.*, “Inferring the molecular and phenotypic impact of amino acid variants with MutPred2,” *Nat. Commun.*, vol. 11, no. 1, Art. no. 1, Nov. 2020, doi: 10.1038/s41467-020-19669-x.

[11] D. Raimondi *et al.*, “DEOGEN2: prediction and interactive visualization of single amino acid variant deleteriousness in human proteins,” *Nucleic Acids Res.*, vol. 45, no. Web Server issue, pp. W201–W206, Jul. 2017, doi: 10.1093/nar/gkx390.

[12] Y. Choi and A. P. Chan, “PROVEAN web server: a tool to predict the functional effect of amino acid substitutions and indels,” *Bioinformatics*, vol. 31, no. 16, pp. 2745–2747, Aug. 2015, doi: 10.1093/bioinformatics/btv195.

[13] E. V. Davydov, D. L. Goode, M. Sirota, G. M. Cooper, A. Sidow, and S. Batzoglou, “Identifying a High Fraction of the Human Genome to be under Selective Constraint Using GERP++,” *PLoS Comput. Biol.*, vol. 6, no. 12, p. e1001025, Dec. 2010, doi: 10.1371/journal.pcbi.1001025.

[14] A. Siepel *et al.*, “Evolutionarily conserved elements in vertebrate, insect, worm, and yeast genomes,” *Genome Res.*, vol. 15, no. 8, pp. 1034–1050, Aug. 2005, doi: 10.1101/gr.3715005.

[15] K. S. Pollard, M. J. Hubisz, K. R. Rosenbloom, and A. Siepel, “Detection of nonneutral substitution rates on mammalian phylogenies,” *Genome Res.*, vol. 20, no. 1, pp. 110–121, Jan. 2010, doi: 10.1101/gr.097857.109.

[16] M. Garber, M. Guttman, M. Clamp, M. C. Zody, N. Friedman, and X. Xie, “Identifying novel constrained elements by exploiting biased substitution patterns,” *Bioinformatics*, vol. 25, no. 12, pp. i54–i62, Jun. 2009, doi: 10.1093/bioinformatics/btp190.

[17] N. Malhis, M. Jacobson, S. J. M. Jones, and J. Gsponer, “LIST-S2: taxonomy based sorting of deleterious missense mutations across species,” *Nucleic Acids Res.*, vol. 48, no. W1, pp. W154–W161, Jul. 2020, doi: 10.1093/nar/gkaa288.

[18] S. Kim, J.-H. Jhong, J. Lee, and J.-Y. Koo, “Meta-analytic support vector machine for integrating multiple omics data,” *BioData Min.*, vol. 10, p. 2, Jan. 2017, doi: 10.1186/s13040-017-0126-8.

[19] H. Qi *et al.*, “MVP predicts the pathogenicity of missense variants by deep learning,” *Nat. Commun.*, vol. 12, no. 1, Art. no. 1, Jan. 2021, doi: 10.1038/s41467-020-20847-0.

[20] K. A. Jagadeesh *et al.*, “M-CAP eliminates a majority of variants of uncertain significance in clinical exomes at high sensitivity,” *Nat. Genet.*, vol. 48, no. 12, Art. no. 12, Dec. 2016, doi: 10.1038/ng.3703.

[21] I. IONITA-LAZA, K. MCCALLUM, B. XU, and J. BUXBAUM, “A SPECTRAL APPROACH INTEGRATING FUNCTIONAL GENOMIC ANNOTATIONS FOR CODING AND NONCODING VARIANTS,” *Nat. Genet.*, vol. 48, no. 2, pp. 214–220, Feb. 2016, doi: 10.1038/ng.3477.

[22] Q. Lu, Y. Hu, J. Sun, Y. Cheng, K.-H. Cheung, and H. Zhao, “A Statistical Framework to Predict Functional Non-Coding Regions in the Human Genome Through Integrated Analysis of Annotation Data,” *Sci. Rep.*, vol. 5, no. 1, Art. no. 1, May 2015, doi: 10.1038/srep10576.

[23] H. A. Shihab *et al.*, “An integrative approach to predicting the functional effects of non-coding and coding sequence variation,” *Bioinforma. Oxf. Engl.*, vol. 31, no. 10, pp. 1536–1543, May 2015, doi: 10.1093/bioinformatics/btv009.

[24] C. Li, D. Zhi, K. Wang, and X. Liu, “MetaRNN: differentiating rare pathogenic and rare benign missense SNVs and InDels using deep learning,” *Genome Med.*, vol. 14, no. 1, p. 115, Oct. 2022, doi: 10.1186/s13073-022-01120-z.

[25] H. Gao *et al.*, “The landscape of tolerated genetic variation in humans and primates,” *Science*, vol. 380, no. 6648, p. eabn8153, Jun. 2023, doi: 10.1126/science.abn8197.

[26] P. Rentzsch, D. Witten, G. M. Cooper, J. Shendure, and M. Kircher, “CADD: predicting the deleteriousness of variants throughout the human genome,” *Nucleic Acids Res.*, vol. 47, no. D1, pp. D886–D894, Jan. 2019, doi: 10.1093/nar/gky1016.

[27] D. Quang, Y. Chen, and X. Xie, “DANN: a deep learning approach for annotating the pathogenicity of genetic variants,” *Bioinformatics*, vol. 31, no. 5, pp. 761–763, Mar. 2015, doi: 10.1093/bioinformatics/btu703.

[28] K. E. Samocha *et al.*, “Regional missense constraint improves variant deleteriousness prediction.” bioRxiv, p. 148353, Jun. 12, 2017. doi: 10.1101/148353.

[29] N. M. Ioannidis *et al.*, “REVEL: An Ensemble Method for Predicting the Pathogenicity of Rare Missense Variants,” *Am. J. Hum. Genet.*, vol. 99, no. 4, pp. 877–885, Oct. 2016, doi: 10.1016/j.ajhg.2016.08.016.

[30] C. Dong *et al.*, “Comparison and integration of deleteriousness prediction methods for nonsynonymous SNVs in whole exome sequencing studies,” *Hum. Mol. Genet.*, vol. 24, no. 8, pp. 2125–2137, Apr. 2015, doi: 10.1093/hmg/ddu733.

[31] N. Alirezaie, K. D. Kernohan, T. Hartley, J. Majewski, and T. D. Hocking, “ClinPred: Prediction Tool to Identify Disease-Relevant Nonsynonymous Single-Nucleotide Variants,” *Am. J. Hum. Genet.*, vol. 103, no. 4, pp. 474–483, Oct. 2018, doi: 10.1016/j.ajhg.2018.08.005.

[32] B.-J. Feng, “PERCH: a unified framework for disease gene prioritization,” *Hum. Mutat.*, vol. 38, no. 3, pp. 243–251, Mar. 2017, doi: 10.1002/humu.23158.
